# Supplementary material for: Are carbon emissions trading and green financial instruments synergistic? -Comprehensive quantitative research based on content analysis
Source: PLoS One. 2024 Mar 7;19(3):e0298601. doi: 10.1371/journal.pone.0298601 (PMC10919601; doi:10.1371/journal.pone.0298601)
Supplement: S1 Data — (ZIP) [file pone.0298601.s001.zip › Data/地级市Z_F工作报告.zip!/─■╧─/╬Γ╓╥╩╨/└┤╫╘╣1⁄2╓┌║┼í╛┬φ┐╦╩2╛▌═°í┐_2017.docx]

各位代表：

现在，我代表市人民政府，向大会报告政府工作，请予审议，并请市政协委员和其他列席人员提出意见。

2016年和本届政府工作回顾

2016年，面对持续加剧的经济下行压力和日益繁重的改革发展任务，在自治区党委、政府和市委的正确领导下，在市人大、政协的监督支持下，全市上下深入贯彻党的十八大、十八届三中、四中、五中、六中全会精神，全面落实习近平总书记视察宁夏重要讲话要求，全力做好稳增长、调结构、促改革、惠民生、防风险各项工作，实现了“十三五”良好开局。预计完成地区生产总值436亿元，增长8.5 %左右；全社会固定资产投资770亿元，增长13%左右；规上工业增加值170亿元，增长9%以上；一般公共预算收入34.4亿元，增长7.8%；社会消费品零售总额104亿元，增长8%；城乡居民人均可支配收入分别为23385元和9975元，增长8.5%和9%。

综合施策精准有效。认真贯彻落实中央、自治区保投资、稳增长和推进供给侧结构性改革等决策部署，结合吴忠实际，加强分析研判，建立“1+5+25+N”统计协调联动新机制，及时研究出台“工业十条”、“农业十条”、“创业就业十条”、“房地产十条”、“十大市场十条”、“旅游业十条”、“特色街区七条”等务实管用的政策措施，促进经济平稳增长。综合运用财税、金融、帮扶等手段，帮助企业克服困难。全面落实市级领导联系包抓重大项目、重点工作“6+6”机制，建立吴忠金超助贷基金等平台，实现担保贷款35亿元。全面推开“营改增”试点，为企业减免税费2.5亿元。中小企业通过股权市场直接融资13亿元。在一系列政策措施的有力助推下，全市经济运行呈现稳中有进、进中向好的良好态势，主要经济指标增速位居全区五市前列。

项目拉动持续有力。坚持重大项目带动，成功签订新疆特变电工宁夏光伏装备制造、哈纳斯500万吨液化天然气一体化基地、昊华骏化清洁能源及城市功能一体化节能综合利用3个投资过百亿元、12个投资过10亿元的大项目。实施招商引资项目275个，到位资金610亿元，落实上争资金140.3亿元。实施PPP项目7个，获得自治区PPP项目综合奖，热电联产供热管网、城市东部地下综合管廊等3个项目被列为国家第三批PPP示范项目。投资结构持续优化，地方投资占固定资产投资90%，民间投资占比达到70%，分别增长6.5%和5.7%。

产业结构不断优化。持之以恒调结构、转方式，盛源食品科技产业园、中国自动化产业园等52个项目建成投产。纺纱、家纺面料产能达到75万锭和1亿米。顺利通过“中国制造2025”试点示范城市评审。新能源建成及在建规模937万千瓦，占全区65%。瓜菜、有机枸杞、酿酒葡萄、中药材种植面积分别达到55万亩、14.5万亩、20.7万亩、50万亩。奶牛存栏21万头，养殖规模化率达到95.4%，高于全国平均水平45.4个百分点。肉牛和肉羊饲养量稳步增长。“盐池滩羊”被指定为G20杭州峰会专用食材，品牌价值68.9亿元。发展硒产品加工企业27家，建设富硒农产品基地13个，我市荣获“中国塞上硒都”称号。万达广场等商业综合体加快建设，鑫鲜农副产品批发市场等“十大市场”业态达到80%以上。房地产开发投资57.8亿元，增长43.3%，销售150.7万平方米，增长55%，去库存周期全区最短。积极打造全域旅游，实施三年行动计划，108塔观景平台建成运营，“印象黄河·彩虹之上”等旅游项目快速推进，全年接待游客564万人次，实现旅游收入29.4亿元，分别增长39%和34%。

城乡建设内涵提升。实施城乡建设项目171个，完成投资140亿元。吴忠热电联产项目点火运行，实现热电供热776万平方米，城区电网入地16.7公里。全力打造餐饮特色小吃街、茶茗文化街、贺兰山东麓葡萄酒风土街、文玩街、健康养生体验街等6条特色街区。建设城市休闲绿道94公里、市政道路30.4公里。新建棚户区改造房7226套。建设美丽小城镇6个、美丽村庄示范点40个、农村公路562公里。完成城市供水水质提标改造工程，备用水源地勘探勘测顺利推进，群众饮水安全得到切实保障。同心下马关等7座大中型调蓄水库加快建设，农田水利基本建设全区“黄河杯”竞赛十连冠。在全区率先制定实施环境保护全面量化管理体系，中央第八环保督察组转办事项全部按时办结。启动实施“蓝天碧水·绿色城乡”专项行动，淘汰停用燃煤锅炉130台，黄标车1589辆，空气质量优良天数同比增加15天。顺利通过国家卫生城市评估验收，成功跻身国家卫生城市行列。

改革开放深入推进。启动119项改革任务。全面推进不动产登记和农村土地承包经营权确权颁证工作。利通区、青铜峡市被评为全国第三届土地节约集约模范县。实施机关公务用车改革，市区公交体制改革全面完成，国有大公交全线运营。组建“八大投”，形成国资国企新布局，实现平台融资14.3亿元，全市国有资产总额增加28亿元。与交通银行、华夏银行签订战略合作协议，新增银行机构10家。嘉泽发电主板上市进入首次公开募股（IPO）排队序列。实施开放吴忠十大行动，万绨旎服饰等企业在国外新设20多个分销点，涝河桥等牛羊肉出口30多个国家。优质特色产品“挺进大中城市”战略加快推进，北京、上海、天津、南京、杭州、沈阳、昆明、福州、贵阳、厦门、大连、宁波、汕头、晋江、义乌15个展示展销中心投入运营。成功举办2016年全国沙滩排球巡回赛，第五届“黄河金岸”国际马拉松赛首次在央视五套全程直播。与中国电子科技集团签订战略框架协议，合作建设智慧城市。青铜峡市被授予“中国西部轴承产业基地”称号。吴忠仪表智能控制阀制造数字化工厂列入国家智能试点示范项目。

民生保障坚实有力。建立脱贫攻坚全面量化管理体系、精准扶贫信息管理平台。建立全区首个建档立卡贫困户评级授信系统，设立风险补偿及产业担保基金7.5亿元，撬动扶贫信贷50.5亿元。全面推行“脱贫保”，创新特色农业等12个险种。实现低保线与扶贫线“两线合一”。脱贫销号65个村，减少贫困人口5万人。城镇新增就业2.7万人，农村劳动力转移就业24.4万人。城乡居民基本养老保险、医疗保险参保超额完成目标任务。城乡低保提标扩面，居民基础养老金人均达到150元。深入推进公立医院综合改革，积极构建分级诊疗体系，全市公立医院药品实行零差率销售。市人民医院新院投入运营，与北京友谊医院等9家知名医疗机构开展务实合作。认真落实全面两孩政策，计划生育、妇女儿童事业得到加强，残疾人、红十字会、慈善事业健康发展，吴忠市位列全国慈善城市百强榜第17位。实施薄弱学校改造工程，改扩建29万平方米，教育教学质量稳步提升，全市高考一、二本上线率达到28.58%。开展文化惠民演出246场，95个公共体育场馆免费向社会开放。新闻出版、广播电视、文史、档案事业进一步发展，作用不断增强。制定实施安全生产全面量化管理体系，全市各类事故起数同比下降54%。启动“七五”普法，深化“平安吴忠”建设，社会治安持续稳定。加大矛盾纠纷排查化解力度，信访批次和人数分别下降16%和32%。加强食品药品监管，食药安全得到有效保障。积极推动军民深度融合发展，我市第三次获得全国双拥模范城称号，青铜峡市第六次获得全国双拥模范城（县）称号。外事、住房公积金、应急管理、防震减灾、气象等工作都取得新成绩。

自身建设得到加强。坚持把纪律和规矩挺在前面，牢固树立政治意识、大局意识、核心意识、看齐意识，重大问题集体研究、向市委报告。认真执行人大及其常委会的决议、决定，主动接受人大工作监督、法律监督，自觉接受政协民主监督，审议、协商事项94项，办结人大代表议案建议69件、政协提案204件。主动听取民主党派、工商联、无党派人士意见建议，支持工会、共青团、妇联等人民团体工作。健全完善政府党组中心组学习制度，建立市长碰头会制度。严格落实从严治党各项规定，自觉执行党风廉政建设责任制，加强财政、审计监督，勤政廉政建设取得新成效，全区群众评议机关和干部作风活动五市排名第一。

各位代表， 2016年是本届政府任期的最后一年。过去四年，是吴忠发展的关键时期，也是困难较多的时期。我们既要应对加快区域发展与脱贫攻坚的双重挑战，又要肩负新常态下稳增长与保民生的双重任务。在这四年里，我们主动适应新常态，积极应对新变化，妥善解决新问题，在转变发展方式中化解经济下行压力，在聚焦精准脱贫中破解发展难题，在深化改革开放中激发内生动力，在苦干实干中努力强市富民，开创了各项事业发展新局面。

四年来，我们坚持加快发展，稳增长、扩投资，努力夯实吴忠的发展基础。全市地区生产总值是2012年的1.4倍，年均增长8.4%。全社会固定资产投资是2012年的2.1倍，年均增长19.2%。一般公共预算收入是2012年的1.2倍，年均增长4.6%。社会消费品零售总额是2012年的1.5倍，年均增长11%。城乡居民人均可支配收入分别是2012年的1.4倍和1.5倍，年均增长8.7%和9.9%。金融机构各项存款、贷款余额突破600亿元和500亿元大关。公共财政预算收入、全社会固定资产投资、金融资源总量跃居全区五市第二。利通区经济总量升至全区第6位，红寺堡区主要经济指标实现翻番，青铜峡市克服下行压力实现稳步发展，盐池县综合实力领跑山区县，同心县跃居山区九县第三。

四年来，我们坚持转型升级，调结构、增效益，努力培育吴忠的产业优势。一二三次产业由2012年的14.9:54.3:30.8调整到12.6:56.4:31。轻工业占比达到30%，高于全区11个百分点。规上工业企业发展到近400户，地方工业比重提升到60.5%。万元GDP综合能耗降幅居全区首位。利通区现代纺织产业园被列入“全国纺织产业转移试点园区”，荣获“纺织结构调整突出贡献奖”。优质粮食、草畜产业、酿酒葡萄规模位居全区第一，特色农业占农业总产值的90%以上。培育农业产业化重点龙头企业202家、新型经营主体2052个。认证“三品一标”农产品244个，建成自治区现代农业示范基地22个。吴忠国家农业科技园区成为全区首个国家有机农产品生产基地。红寺堡区荣获“中国最具发展潜力葡萄产区”称号，利通区被确定为第一批“国家农产品质量安全县创建试点单位”，同心县被评为国家级有机枸杞出口食品农产品质量安全示范区。打造健康产业新业态，健康产业增加值占GDP的10%以上。盐池县、同心县、红寺堡区、青铜峡市进入国家电商进农村示范县行列，建成县级服务中心6个、村级服务站223个、培育电商运营企业50余家。盐池县成功举办全国跳伞锦标赛，红寺堡区连续承办四届全国青少年航空航天模型锦标赛。“黄河大峡谷·中华黄河坛”跻身国家4A级景区。

四年来，我们坚持以人为本，强基础、增内涵，努力提升吴忠的城镇功能。累计投资761亿元，实施城乡建设项目1416个，全市城镇化率达到55.3%，年均提高2个百分点。利青一体、银吴同城进程加快，城市建成区扩展到125平方公里。综合改造老旧小区157个，完成老旧小区既有建筑节能改造550万平方米。银西高铁、吴忠至中卫城际铁路、京藏高速改扩建等重大交通项目开工建设，罗山大道等一批等级公路建成通车，公路通车总里程达到8648公里，行政村公路通达率100%。红寺堡区、盐池县、同心县城市发展空间进一步拓展。建设美丽小城镇24个、美丽村庄128个。全市森林覆盖率达到15.5%，城市建成区绿地率、绿化覆盖率、人均公园绿地面积分别达到34.6%、35.7%和15.9平方米，黄河吴忠段水质稳定保持Ⅱ类优水质，空气质量优良天数位居全区沿黄城市前列。先后荣获全国绿化模范城市、中国人居环境范例奖、中国十佳绿色城市等荣誉称号，成为全区唯一的国家生态文明建设试点市。

四年来，我们坚持改革开放，破瓶颈、求创新，努力释放吴忠的发展活力。我市被列为全国中小城市综合改革试点市，率先在全区公布实行“两个清单”，商事制度改革走在全国前列，市场主体总量跃居全区五市第二。成为全区唯一实现村镇银行县区全覆盖的地级市，被确定为全国31个小微企业信用体系建设试验区之一。10家企业在新三板上市，45家企业在区域性股权交易中心挂牌。“宽带中国”示范城市建设走在全国前列，电信普遍服务网络覆盖率达90%以上。青铜峡市五星村土地股份合作社成为全区首家农民股份合作经营实体。医药卫生、教育等领域改革取得重要进展。金融扶贫“盐池模式”、文化市场“执法+协会”监管模式在全国推广。培育国家级高新技术企业3家，自治区级技术创新中心等研发平台30个。成功举办两届“黄河金岸”国际马拉松赛等赛事节会，吴忠的影响力和美誉度进一步提升。与北京西城区、天津武清区、上海青浦区、广西崇左市建立友好城市关系。累计争取资金505亿元，招商引资2225亿元。外贸进出口总额年均保持两位数增速。

四年来，我们坚持民生为先，兜底线、促公平，努力增进吴忠的百姓福祉。每年公共财政支出的83%以上用于改善民生。全面小康指标中人民生活小康实现程度由77.8%提高到86.2%。四年办理民生实事46件。搬迁安置移民1.57万户7.03万人，圆满完成“十二五”生态移民任务。减少贫困人口13.2万人，贫困发生率下降13.5个百分点。新建、改建学校425所，在全区率先实现基本普及高中阶段教育目标，利通区、青铜峡市进入全国义务教育发展基本均衡县（市、区）行列，同心县被评为全国农村学生营养改善计划工作先进县。我市成功入选全国百城技能振兴专项活动城市。农村标准化村卫生室实现全覆盖，“15分钟医疗卫生服务圈”基本形成。大病医保实现全覆盖。为10多万被征地农民办理养老保险，解除后顾之忧。建设保障性住房59133套，12万住房困难群众喜迁新居。实施了一批民生水利工程，60多万农村群众饮水安全问题得到解决。积极创建全国文明城市，成功获批创建第三批国家公共文化服务体系示范区，两次荣获全国七星级慈善城市。

四年来，我们坚持依法行政，转作风、提效能，努力维护吴忠的和谐稳定。办理人大代表议案建议311件、政协委员提案846件。深入推进依法治市，我市荣获全国法治城市创建活动先进单位，盐池县连续三次荣获全国法治宣传教育先进县。“六五”普法全面完成，法治观念深入人心。成立吴忠市决策咨询委员会，聘请127位知名专家学者担任法治建设、社会管理、产业发展等14个领域的政府顾问，其中“两院”院士15名。深入开展群众路线教育实践活动、“三严三实”专题教育和“两学一做”学习教育，各级干部能力素质明显提升。连续三年获得全区效能目标考核一等奖。坚决执行中央和自治区关于改进作风、密切联系群众各项规定，行政监察和审计监督进一步加强，工作作风持续好转，发展环境进一步优化，连续两年全区群众评议机关和干部作风活动五市排名第一。安全生产、信访工作考核连续三年全区第一。鲁忠义家庭荣获“全国文明家庭”称号，王金香家庭荣获“全国最美家庭”称号，刘志海等7人荣登“中国好人榜”，赵峰等3人荣获“感动宁夏年度人物”。成功创建为“全国民族团结进步示范市”，五个县（市、区）全部被命名为“全国民族团结进步示范县”。

四年的丰富实践，使我们深深体会到：必须始终坚持党的领导，依法行政。一切以党和人民事业为重，与党中央、自治区党委和市委保持高度一致，以党的建设为统领推动政府各项工作，依法全面履行政府职能。必须始终坚持发展第一，民生为先。面对发展不足的基本市情，始终做到不动摇、不懈怠、不折腾，聚精会神搞建设，一心一意谋发展。坚持以百姓之心为心，把办好人民群众的事情作为一切工作的出发点和落脚点，顺民意、暖民心、释民怨、解民困，汇聚民智，改善民生。必须始终坚持立足实际，发挥优势。坚持特色化发展，充分发挥吴忠在资源、人文、产业等方面的比较优势，扬长避短、取长补短，努力扩优势、强特色、补短板，走出一条特色充分凸显、优势不断释放的转型发展之路。必须始终坚持改革创新，扩大开放。面对制约和影响经济社会发展的诸多矛盾问题，以改革的思维、创新的精神、开放的姿态，坚决破除体制机制障碍，敢于触及深层次矛盾问题，为经济社会持续健康发展提供新动力，开辟新空间。必须始终坚持从严管理，改进作风。牢记宗旨，不忘初心，坚定不移地贯彻执行党的群众路线，从严教育、从严要求、从严监督，各级干部真抓实干，全市上下携手奋进，汇聚推动发展的强大合力。

各位代表，回顾本届政府四年的工作，我们遇到的困难比预想的大，取得的成绩比预期的好。这是自治区党委、政府正确领导的结果，是市委总揽全局、科学谋划的结果，是人大、政协有力监督、大力支持的结果，是全市人民精诚团结、奋力拼搏的结果，也是一任接着一任干，绵绵用力、久久为功的结果。在此，我代表市人民政府，向奋战在各条战线的广大干部群众，向人大代表、政协委员、各民主党派、工商联，向离退休老同志和社会各界人士，向驻吴人民解放军、武警官兵、政法干警，向中央及区属驻吴单位，向关心支持吴忠发展的国家部委、自治区厅局、友好城市、政府顾问和外商、港商、台商表示崇高的敬意和衷心的感谢！

同时我们也清醒地认识到，全市经济发展水平还比较低，经济总量不大、发展不足仍是最大市情；产业结构性问题依然突出，传统产业改造升级力度不大，新兴产业拉动能力有待提升，转型升级仍面临不少困难；城乡、山川发展不平衡，公共服务水平还不高，脱贫攻坚任务十分艰巨；财政收支矛盾突出，城乡居民收入总体水平偏低，保障改善民生需要付出更多努力；维护社会和谐稳定面临许多新情况新问题，社会治理创新有待进一步加强；政府职能转变还不到位，一些干部干事创业劲头不足、工作落实力度不够等问题还不同程度存在，等等。对这些问题，我们必须认真对待，采取有力措施，切实加以解决。

今后五年规划目标和主要任务

未来五年，是全面建成小康社会的决胜时期，也是实现吴忠全面转型发展的关键时期，更是确保如期实现脱贫目标的攻坚时期。市第五次党代会确定了坚持绿色发展、推动转型升级、全面建成小康社会的奋斗目标。我们要坚持新发展理念，明确新方位、聚集新动能、释放新活力，加快转型升级，实现弯道超车。

今后五年政府工作的总体要求是：高举中国特色社会主义伟大旗帜，以邓小平理论、“三个代表”重要思想、科学发展观为指导，全面贯彻党的十八大、十八届三中、四中、五中、六中全会精神，深入贯彻习近平总书记系列重要讲话精神，统筹推进“五位一体”总体布局，协调推进“四个全面”战略布局，自觉践行“五大发展理念”，认真落实自治区党委、政府及市委决策部署，以绿色发展、转型升级为主题，以提高发展质量和效益为中心，率先走出西部欠发达地区绿色发展新路子，建设绿色经济示范市，全面建成小康社会。

主要预期目标是：地区生产总值、全社会固定资产投资、规上工业增加值、一般公共预算收入年均保持8%、12%、9%、8%以上增速，人均GDP接近4万元。城乡居民人均可支配收入年均分别增长8%、9%以上。农村贫困人口全部脱贫，贫困县全部摘帽。完成自治区下达的环境质量改善目标和节能减排任务。

今后五年要在以下六个方面谋求新突破、取得新进展。

构筑绿色产业体系。着力构建以新型工业为主体、现代农业为基础、现代服务业为支撑的绿色产业体系，推动产业向中高端迈进。实施工业转型升级行动计划，大力发展绿色工业，全力构建食品工业、装备制造、油气化工、生态纺织、新能源、煤化工、新材料、葡萄酒、生物制品、煤炭电力10个“百亿元”板块和食品工业、健康产业2个“千亿元”产业，着力打造吴忠经济“升级版”。抢抓创建“中国制造2025”试点示范城市机遇，实施质量立市和品牌兴吴战略，打造西部制造业强市。积极发展新能源产业，建成国家新能源综合示范区。全市工业总产值突破千亿元大关，轻重工业比达到35:65。实施农业提质增效行动计划，大力发展绿色农业，推动优质粮食、有机枸杞、酿酒葡萄、滩羊等优势特色产业精品化、高端化，打响绿色、生态、有机、富硒品牌。打造孙家滩生态农业“有机谷”。农业总产值突破140亿元。实施服务业融合发展行动计划，大力发展绿色服务业，推动文化旅游、电子商务、金融保险等融合发展。实施文化旅游融合发展行动计划，构建多元化旅游产品体系，形成“两区两带”旅游产业格局。现代服务业占第三产业比重达60%以上。

推进城乡一体发展。秉承生态、智慧、人文、低碳理念，深入推进新型城镇化建设。主动融入大银川都市区，加快利青一体、银吴同城步伐，打造银吴“半小时经济圈”。推进银西高铁、城际铁路等重大交通项目建设，增强区域交通区位优势。全市城镇化率达到60%以上。推广应用绿色建筑和节能技术，力争城镇绿色建筑比重达到50%以上。争创全国可再生能源示范城市。实施中部干旱带大中型水库工程，完善城市防洪排涝体系。围绕产业特色和地域文化，统筹推进盐同红大县城和美丽乡村建设，集中建设一批特色城镇和美丽村庄，美丽乡村覆盖面达到90%以上。实施农村环境治理行动计划，打造美丽宜居幸福新农村。加快建设智慧吴忠，建成城市数字综合管理运行平台，让群众在智慧城市里生活得更美好。探索环卫保洁、园林绿化等领域市场化运作模式，提升城市精细化管理水平。

打造吴忠靓丽名片。大力推进生态文明建设，打造天蓝地绿、山清水秀、空气清新、环境宜人的美丽吴忠。实施生态绿化美化行动计划，全市森林覆盖率力争达到18%以上，争创国家森林城市，让绿色成为吴忠的底色。加快连通环城水系，创造更多湿地资源，为城市提供更多的“绿肺”空间，增添吴忠的灵气。启动环境质量保障立法，守住底线，不碰红线，保护好绿水青山。完善资源有偿使用和生态补偿机制，做到源头严防、过程严管、后果严惩。积极开展节能量、碳排放权和排污权交易试点。认真落实环境保护全面量化管理体系，推动政府环保职能履行到位。建成国家生态文明建设示范市、国家环保模范城市、国家低碳试点城市。空气质量优良天数比例达到85%以上。

全面深化改革开放。全面取消非行政许可审批事项，落实清单管理制度，实现全市联网审批。做大做强八大投资公司，健全国资监管体制，提高国有资本运营效率。筹建吴忠银行，构建多元金融服务体系。引导农村土地市场化、集约化、资本化运作，争取设立面向全区全国的农村产权交易中心。深化区域协作，打造承接产业转移示范区。加快实施优质特色产品“挺进大中城市”战略，支持企业组团发展。加快科技创新，完善后补助机制及成果转化机制，建设一批国家和自治区级重点实验室、企业技术中心、院士工作站、博士工作站，力争金积工业园、吴忠国家农业科技园进入国家高新技术产业园区行列。深化与国内知名科研院校合作，不断提升产学研融合水平。到2020年，全社会研究与试验发展经费(R&D)占GDP比重达到2.1%，科技进步贡献率达到55%以上。

持续增进民生福祉。始终坚持民生优先，共建共享。认真落实脱贫攻坚全面量化管理体系，深入开展“五个一批”工程和“十三项”脱贫计划。扩大普惠性幼儿园覆盖率，推进义务教育均衡发展，全面普及高中阶段教育。实施职业教育提升行动计划，建成红寺堡区、同心县职业教育学校，进一步提升职业教育办学水平。大力促进就业创业，多渠道增加群众收入。实施健康产业发展行动计划，深入推进健康吴忠建设。坚持预防为主，加快健康服务、疾病预防和医疗保障体系建设，不断提高公共卫生服务能力。广泛开展全民健身运动和群众性文化活动，高水平承办自治区第十五届运动会，成功创建国家公共文化服务体系示范区。推动社会保险提标扩面，全面落实城乡居民大病医疗保险和大额医疗救助制度。加快构建多层次、多元化养老服务体系。

营造良好发展环境。坚持依法治市、依法行政，切实提高运用法治思维和法治方式推动发展的能力和水平。健全和完善决策程序，推进科学民主决策。加快职能转变，全面准确履行政府职能。依法严厉打击各类违法犯罪活动，深入推进禁毒人民战争，争创全国禁毒示范市。深入开展“七五”普法，教育引导全民自觉守法。全面落实安全生产全面量化管理体系，提高事故应急救援保障能力。深化民族团结进步创建，依法加强宗教事务管理，把我市民族团结、宗教和顺的名片打造得更加靓丽。加强食品药品安全监管，确保群众饮食用药安全。全面推进网格化管理，不断完善基层社会管理和服务体系。加强精神文明建设，争取创建成为全国文明城市。持续加强作风建设，进一步优化发展环境，着力打造高效廉洁政府。

各位代表，坚持绿色发展，加快转型升级，全面建成小康，是时代的要求、人民的期盼，更是我们的庄严承诺。只要我们咬定目标、不忘初心、继续前进，经过五年的努力，一个更有实力、更具活力、更富魅力、更加美丽的吴忠必将展现在世人面前！

2017年预期目标和主要工作

2017年是党的十九大和自治区第十二次党代会的召开之年，是实施“十三五”规划的重要一年，是供给侧结构性改革的深化之年，是全面建成小康社会的关键一年。全市经济社会发展的主要预期目标和约束性指标是：地区生产总值增长8%，全社会固定资产投资增长12%以上，一般公共预算收入同口径增长8%以上，社会消费品零售总额增长8%，城乡居民人均可支配收入分别增长8%、9%，城镇登记失业率控制在4%以内，居民消费价格涨幅控制在3%以内，完成自治区下达的环境质量改善和节能减排任务。

重点抓好以下十个方面的工作。

项目建设再发力。以项目保投资、稳增长、促转型，确保上争项目资金不低于135亿元，招商引资实际到位资金650亿元。加快重点项目建设。实施伊利液态奶四期等506个产业项目，棚户区改造等273个基础设施项目，东片区初级中学等108个社会事业项目，年度完成投资860亿元。拓宽项目资金渠道。启动实施国家中小城市PPP创新工作试点城市创建工作，加快推进水生态治理、红寺堡罗山机场等PPP示范项目，鼓励引导社会资本参与生态环保、农林水利、市政基础设施、社会事业等领域建设，形成多元投资格局。精准对接国家政策导向、投资和产业发展方向，谋划储备一批市场潜力大、投资回报好、发展后劲足的重大项目。以全国工商联十一届十次常委会暨民营企业助推宁夏创新发展大会、中阿博览会为契机，坚持招大引强、招高引新、招才引智，确保一批意向性项目尽快落地。

工业转型再突破。制定实施工业绿色发展规划，深入开展“中国制造2025”试点示范城市创建工作。实施5000万元以上重点工业项目100个，新增规模以上工业企业20户，规模以上工业增加值增长9%以上。培育壮大优势主导产业，加快盛源食品科技园、中民裕丰食品加工、天津春发集团咸味食品香精等项目建设，力争食品工业产业产值增长20%以上。积极引进江苏盛氏、浙江华都等纺织企业入驻现代纺织产业园，确保恒丰、德悦高端纺纱和兴德棉高档家纺面料织造项目投产达效，力争纺织业产值增速达到30%以上，轻重工业比达到31:69。实施新疆特变电工光伏发电暨装备制造配套等项目，推动吴忠仪表等装备制造项目向高端领域拓展。积极推进中民投光伏发电、三峡新能源风电等新能源项目建设。加速传统产业提档升级，加快推进哈纳斯500万吨液化天然气一体化基地等项目建设，力促昊华骏化集团80万吨甲醇、太阳镁业30万吨盾镁电热法镁合金等项目开工建设。积极化解过剩产能，创造条件推动企业兼并重组、行业整合，引导青铝、通达等企业调整产品结构，坚决淘汰煤炭、冶炼等行业落后产能，工业能耗下降3%。大力发展非公有制经济，支持中小企业向“专精特新”方向发展。着力提升园区发展水平，金积工业园区启动运行食品检测中心、研发中心，创建自治区级金融示范园区，争创国家级循环化示范园区。进一步调整优化各工业园区功能定位和产业布局，完善污水处理等基础设施，盘活闲置土地资源，增强园区聚集效应。加强工业经济运行分析调度，深化市级领导精准包抓企业责任制，切实帮助企业解决困难问题。积极争取国家、自治区基金、信贷、运力支持，有效降低企业用电、物流、融资等成本，帮助企业在转型发展中走出新路子、创出新天地。

现代农业再提升。积极推进现代农业产业、生产、经营三大体系建设，力争农业增加值增长4.5%以上。加快农业产业化发展，全市粮食种植面积稳定在320万亩，瓜果蔬菜面积达到95万亩，新增有机枸杞8500亩、黄花菜2万亩。积极推进现代畜牧业全产业链建设，全市奶牛稳定在22万头，新增肉牛5万头、肉羊20万只。加快推进红寺堡肖家窑、青铜峡鸽子山等葡萄种植基地建设，新建酒庄15家。扩大农业规模化经营，鼓励支持龙头企业组建产业联合体，引导农户通过联耕联种、土地流转等多种方式实现连片耕种。全市新增市级以上龙头企业10家、新型经营主体100家。新增富硒农产品生产示范基地10个，富硒农产品种植面积达到10万亩。开展农民职业技能培训，帮助农民持续增收。推动农业标准化生产，率先在奶牛和富硒产业实现标准化生产，引导和推进农产品生产加工向标准化、规范化、品牌化方向发展，增加绿色优质农产品供给。支持龙头企业走精深加工路子，提高附加值，打造高端品牌。大力推广农业新技术、新品种、新机具，农业科技贡献率和农业综合机械化水平分别达到62%和77%。着力夯实农业基础，因地制宜开展农田水利基本建设，建立健全农田水利建设管护长效机制。深入实施灌区续建配套、盐碱地改良、节水灌溉等项目，加快推进盐同红扶贫攻坚水源工程建设，发展高效节水农业8万亩。完善农业社会化服务体系，支持保险机构开展政策性涉农保险业务，加强科学防灾减灾，切实增强农业抗风险能力。完成第三次全国农业普查。

第三产业再融合。推动业态融合互补，力争第三产业增加值增长8.5%以上。延伸发展现代服务业，加快利通区养生健康产业园等项目建设，培育发展中医药、保健养生等业态。推进红寺堡、青铜峡、盐池、同心电商进农村示范县工程，力争农村电子商务覆盖率达到90%以上，争创国家级电子商务示范市。大力发展通航产业、临空经济。依托环境人文优势，吸引举办节会、展览、展销，培育发展会展经济。改造提升传统服务业，推进商贸物流业健康发展，加快万达广场、京东冷链物流节点等大型商贸项目建设。打造物流综合服务平台，建成宁鲁石化物流园等综合性物流中心6个。大力实施餐饮提升工程，制定特色小吃标准，扩大 “吃在吴忠”品牌影响力。融合发展文化旅游业，推进全域旅游示范创建工作。大力发展休闲观光游、乡村生态游、民俗风情游，培育低空旅游、工业旅游等新业态，丰富旅游产品供给，提升吴忠旅游全域化、特色化、品质化水平。启动韦州古城历史文化景区、东部环线旅游风景道等项目，黄河大峡谷·中华黄河坛争创5A级景区。全年游客接待和旅游收入分别增长12%和15%。

城乡建设再提质。加快推动以人为核心的新型城镇化步伐，实施城乡建设项目159个，投资142亿元，城镇化率达到57.3%。拓展优化发展空间，完成吴忠市空间规划编制工作和空间规划试点任务。加快京藏高速公路改扩建等项目建设，实施国道344线吴忠至灵武段、青铜峡至银川正源街快速通道项目，打通向南、向北、向东通道。建成运营黄河文体会展中心。启动银西高铁枢纽站广场、道路、水系、绿化等配套设施建设。深入推进智慧吴忠建设，积极争创新型智慧城市试点，推进城市数据运营中心建设。新增地下综合管廊4.5公里，新建、续建城市道路12条，改造老旧小区10个。建立城市供暖、供水、供气等智能化平台，创新城市管理和卫生保洁机制。加快打造餐饮特色小吃街等6条特色街区，提升城市品味。实施红寺堡第二污水处理厂、盐池东区集中供热、同心清水河县城段综合治理等工程，不断完善县城功能。深化国家级文明乡（村）创建，加快金银滩、惠安堡等5个美丽小城镇，渠口、李岗子等30个美丽乡村建设。新建改建农村公路480公里。加快农村危房危窑改造，彻底解决贫困群众住房安全问题。实施农村环境综合整治，加快推进农村改厕及污水治理，健全农村环境卫生管护长效机制，切实改善农村人居环境。

生态环境再改善。大力实施生态立市战略，使吴忠的天更蓝、水更清、地更绿、空气更清新。加强环境污染防治，扎实推进国家生态文明建设试点市、国家环保模范城市创建步伐，深入开展大气、水、土壤污染防治专项行动，市区全面淘汰燃煤锅炉，确保全年空气质量优良天数达到78%以上。推进污水处理提标扩容，实现稳定运行、达标排放，确保黄河吴忠段水质稳定保持在Ⅱ类优水质。全面开展污染源普查工作，加强城乡集中式饮用水源地保护，加大农村面源污染和工业固废污染治理。启动实施秸秆综合利用、病死畜禽无害化处理项目，推动中科国通生活垃圾焚烧发电、餐厨垃圾无害化处理等项目建成投入使用。坚守耕地红线，开展土地利用效益评价，确保集约利用，保护好引黄灌区精华之地，绝不允许浪费破坏和低效益开发利用。构筑绿色生态屏障，深入实施退耕还林、退牧还草、防沙治沙等项目，全年新增营造林19.3万亩，治理水土流失240平方公里，森林覆盖率达到16%。完善市区街道绿网体系，实施城市绿化提质增景改造工程，提升绿地养护管理水平。加强自然保护区、湿地公园管理，巩固全国湿地产权试点工作成果。建立环保长效机制，严格执行环保“负面清单”，落实环境保护全面量化管理体系。建立领导干部任期生态文明建设责任制，探索开展环境保护审计工作，让终身有责、失职追责成为生态环境保护的“高压线”。全面推行市、县、乡“河长制”。严格化工类项目安全生产和环境影响评价，绝不要带毒发臭的GDP。

改革开放再深入。始终把改革开放贯穿经济社会发展全领域、全过程，不断释放发展活力。着力优化服务环境，积极做好“接放管服”工作，完善“两个清单”动态调整机制，完成“政务云”行政审批系统建设。深入推进企业“五证合一”、个体工商户“两证合一”、“一照一码”登记制度改革，力争市场主体增长8%以上。深化财税金融改革，全力开源挖潜，加大综合治税，力促财税稳健增长。完善全口径债务管理，有效防范政府债务风险。支持金融机构延伸服务网点，年内新设金融分支机构2家以上，新增信贷资金力争达到30亿元。鼓励引导企业通过股权交易等方式直接融资，争取年内10家企业在新三板、区域性股权交易中心挂牌。力争宁夏首家互联网金融机构落户吴忠。深化国有企业改革，充分发挥“八大投”融资平台作用，确保国有资产保值增值。释放农业农村活力，加快“三权分置”农村新型经营体制改革，落实所有权，稳定承包权，放活经营权。加快推进农村宅基地使用权和房屋所有权“两证合一”颁证工作，探索制定农民自愿永久有偿退出“三权”管理办法。积极促成全区农村产权交易中心落户吴忠。扩大集体资产股权量化，土地股份合作制试点范围。鼓励个体工商户和灵活就业人员自主缴存住房公积金，扩大制度覆盖面。全面放开落户限制，有序推进农业转移人口市民化。努力扩大对外开放，深入实施开放吴忠十大行动，深化与京津冀、长三角等地区交流合作，精心承办第十届国际葡萄与葡萄酒学术研讨会。加快重庆、成都、广州等城市优质特色产品展示展销中心建设步伐，年内建成20家。

科技创新再助力。落实科技创新驱动发展战略，全面实施科技创新行动计划。推进产学研融合，围绕高端智能控制阀、滚动轴承、精密汽车零部件，引进培育生产研发类企业，壮大高新技术产业规模。深化与清华大学、中科院、西北农林科技大学等科研院所、国家高新区合作。强化企业创新主体地位，组建产业技术创新战略联盟。启动金积工业园区、吴忠国家级农业科技园区创建国家高新技术产业开发区工作，金积工业园区建成自治区级高新技术开发区。加强智力支撑，围绕项目开发人才，创新用人体制机制。引进装备制造、新材料、生态纺织等领域高层次创业人才（团队）5个，建成院士工作站1个，培养产业领军人才10名，培育科技特派员及农村实用人才带头人300名、经营性人才500名。加快成果转化，鼓励企业加大研发资金投入，扶持本土企业承接科技成果转化，先进技术成果转化达到10项以上。新建自治区级企业技术中心2家，培育“两化融合”示范企业5家，培育科技型中小企业20家以上。加强与广东中山健康产业园合作，引进生物保健、营养食品、中草药种植等产业项目。争创驰（著）名商标、名牌产品5个。

社会事业再发展。加快完善基本公共服务体系，满怀真情办好民生实事，千方百计提高城乡居民收入，努力让群众有更多“获得感”和“幸福感”。深入推进脱贫攻坚，严格落实脱贫攻坚全面量化管理体系，全面推进产业、光伏、电商、金融、旅游、教育扶贫，复制推广金融扶贫“盐池模式”，精准落实到村到户脱贫措施，确保59个贫困村销号，1.3万户5万人脱贫，盐池县率先脱贫摘帽。创新移民安置模式，完善基础设施，年内搬迁安置移民3724人。统筹发展社会事业。加大教育投入力度，免除高中阶段教育学杂费，提高生均公用经费标准。实施宁夏民族职业技术学院产教融合等项目，改扩建各类中小学和幼儿园102所。推动义务教育均衡发展，确保红寺堡区、同心县顺利通过自治区评估验收。加快医药分开、分级诊疗、三医联动，促进基本医疗卫生服务更公平。完成妇幼保健院迁建工程。深化与北京友谊医院等医疗机构的交流合作，让群众享受到更优质、更便捷的医疗服务。加强健康知识普及教育，提高群众健康意识。全面落实两孩政策，提升人口素质。深入推进国家公共文化服务体系示范区创建，确保公共文化服务均等化、社会化发展。高标准举办第三届全市运动会、第六届黄河金岸国际马拉松赛、全国沙滩排球巡回赛和山地自行车赛等体育赛事。大力开展特色技能人才培训，创建一批全民创业孵化基地，重点抓好高校毕业生、农村富余劳动力和困难群体就业，全年培育小企业650个，培养小老板1300个，让每一个诚实劳动者有活干、有钱挣。完善社会保障体系，推进全民参保登记和扩面征缴，启动实施异地就医结算区级统筹。新建棚户区改造房9454套，着力解决城乡低收入家庭住房问题。进一步提高城乡居民基础养老金，加快推进城乡低保一体化，完善社会救助体系，提高困难群众和残疾人生活救助金标准，让困难群众和弱势群体都能感受到社会主义大家庭的温暖。积极发展慈善事业和养老服务产业，年内建成吴忠市敬老院、老年活动中心、残疾人康复中心。建立完善政府、学校、家庭责任共担的关爱管护留守儿童工作机制，动员社会力量参与，让留守儿童得到更多关爱保护。全面强化社会治理，深化“平安吴忠”建设，构建立体化社会治安防控体系。切实加大禁毒工作力度，深化综合整治成效，坚决打好禁毒人民战争。积极做好信访积案化解，严厉打击恶意欠薪行为，保障农民工合法权益。认真落实安全生产全面量化管理体系，加强煤炭、化工、道路、食品等重点领域监管。巩固提升全国民族团结进步示范市创建成果，着力提升宗教事务依法管理水平，引导宗教与社会主义社会相适应。扎实开展全国文明城市创建活动，提高社会文明水平。启动新一轮全国双拥模范城创建工作，巩固深化军民融合发展成果。加强外事侨务、气象、统计、档案、市志、防震、住房公积金等工作，支持工会、共青团、妇联等群众团体创新开展工作。

自身建设再加强。面对艰巨繁重的发展任务，政府部门和全体工作人员要按照“实干、团结、创新、高效、清廉”的要求，恪尽职守，努力打造人民满意政府。始终坚持党的领导，牢固树立政治意识、大局意识、核心意识、看齐意识，始终同以习近平同志为核心的党中央保持高度一致，坚决贯彻中央和自治区的决策部署，紧紧围绕市委确定的战略、目标、任务狠抓落实。坚持从严管理，认真落实“三严三实”要求，不断加强政府及组成部门党组思想建设、政治建设、组织建设和作风建设。始终坚持依法行政，严格按法定权限和程序履行职责，切实做到法定职责必须为，法无授权不可为。自觉接受人大及其常委会依法监督和政协民主监督，提高议案建议、提案办理质量。深入推进政务公开，及时回应社会关切，提高政府工作透明度。始终坚持务实勤政，大力倡导说了就办、定了就干、干就干成。健全督查问责、激励鼓励和容错纠错机制，整肃庸政懒政怠政行为，给改革创新者撑腰鼓劲，让守旧不前者无处立足，持续提高政府执行力、服务力和保障力。始终坚持从严治政，把纪律和规矩挺在前面，严格落实中央“八项规定”精神，认真履行“一岗双责”，持之以恒改进作风，严肃查处违纪违法案件，坚决铲除滋生腐败土壤。坚持厉行节约，真正把财政资金用到发展最需要、群众最受益的地方。政府工作人员要自觉接受纪律的约束和舆论的监督，干净干事，清白做人，做无愧于组织、无愧于人民、无愧于时代、无愧于使命、无愧于人生的人民满意的公务员。

各位代表，新目标振奋人心，新使命催人奋进。让我们更加紧密地团结在以习近平同志为核心的党中央周围，在自治区党委、政府和市委的坚强领导下，改革创新，开拓进取，勇于担当，奋发有为，努力走好新的长征路上的每一步，加快建设“五个吴忠”，为实现经济繁荣、民族团结、环境优美、人民富裕，与全国同步建成全面小康社会目标而努力奋斗，以优异成绩迎接党的十九大和自治区第十二次党代会的胜利召开！
